# RETRACTED: Hindi, S.S. Novel Recycling, Defibrillation, and Delignification Methods for Isolating α-Cellulose from Different Lignocellulosic Precursors for the Eco-Friendly Fiber Industry. *Polymers* 2024, *16*, 2430

**DOI:** 10.3390/polym18111353

**Published:** 2026-05-29

**Authors:** Sherif S. Hindi

**Affiliations:** Department of Agriculture, Faculty of Environmental Sciences, King Abdulaziz University (KAU), P.O. Box 80208, Jeddah 21589, Saudi Arabia; sshindi166@gmail.com; Tel.: +966-566760086

The journal retracts the article titled “Novel Recycling, Defibrillation, and Delignification Methods for Isolating α-Cellulose from Different Lignocellulosic Precursors for the Eco-Friendly Fiber Industry” [1], cited above.

Following publication, concerns were raised with the Editorial Office regarding the integrity of the images and the scientific accuracy of the data presented in the paper [1].

Adhering to our standard procedure, the Editorial Office and Editorial Board conducted an investigation that identified indications of inappropriate image editing and duplication in Figure 4b,j [1] and confirmed inconsistencies within the data presented in Figures 9 and 10 of this article [1]. While the author cooperated in the investigation, raw data meeting the journal’s requirements could not be provided for Editorial Board evaluation. Consequently, the Editorial Board has lost confidence in the reliability of the findings and has decided to retract this publication, as per MDPI’s retraction policy.

This retraction was approved by the Editor-in-Chief of the *Polymers* journal.

The author disagreed with this retraction.
